# Supplementary material for: The Comparison of the Effects between Continuous and Intermittent Energy Restriction in Short-Term Bodyweight Loss for Sedentary Population: A Randomized, Double-Blind, Controlled Trial
Source: Int J Environ Res Public Health. 2021 Nov 5;18(21):11645. doi: 10.3390/ijerph182111645 (PMC8583133; doi:10.3390/ijerph182111645)
Supplement: Supplementary file 1 [file ijerph-18-11645-s001.zip › The template of detailed self-recorded report summaries.pdf]

Name	Group	Gender	Age	Body Height (cm)	Baseline Bodyweight (kg)	Endpoint Bodyweight (kg)	Bodyweight Difference (kg)	Baseline BMI (kg/m^2)	Endpoint BMI (kg/m^2)	BMI Difference (kg/m^2)	Baseline RMR (kcal/day)	Endpoint RMR (kcal/day)	RMR Difference (kcal/day)

Total Carbonhydrate Intake (g)	Total Protein Intake (g)	Total Fat Intake (g)	Daily Energy Intake (kcal)	Total EAT (kcal)	Energy Restriction (kcal/day)
Percentage				Average Daily EAT (kcal/day)	Exercise Requirement
			100%		
Average Daily Intake (g/day)				Average Daily Energy Deficit (kcal/day)	Other Information

Day	Carbonhydrate (g)	Protein (g)	Fat (g)	Daily Energy Intake (kcal)	EAT (kcal)	Energy Deficit (kcal)
1						
2						
3						
4						
5						
6						
7						
8						
9						
10						
11						
12						
13						
14						
15						
16						
17						
18						
19						
20						
21						
22						
23						
24						
25						
26						
27						
28						
